# An Efficient and Robust Ellipse Detection Method for Spacecraft Docking Rings in Complex Scenes

**DOI:** 10.3390/s26020396

**Published:** 2026-01-07

**Authors:** Qi Wu, An Shu, Haodong Pei, Kun Yu, Muyun Luo, Yunmeng Liu

**Affiliations:** 1 Shanghai Institute of Technical Physics, Chinese Academy of Sciences, Shanghai 200083, China; wuqi23@mails.ucas.ac.cn (Q.W.); yukun@mail.sitp.ac.cn (K.Y.); luomuyun23@mails.ucas.ac.cn (M.L.); lym_sitp@163.com (Y.L.); 2 University of Chinese Academy of Sciences, Beijing 100049, China; 3 Shanghai Integrated Innovation Center for Space Optoelectronic Perception, Shanghai 200083, China

**Keywords:** non-cooperative target, ellipse detection, quadrant division, arc pruning, on-orbit service

## Abstract

The key components of spacecraft are typically present as circular or near-circular structures, and their precise and rapid extraction is essential for spacecraft attitude estimation. In response to the high precision and robust detection of ellipse components on space non-cooperative targets such as spacecraft docking rings, this paper proposes an efficient and robust ellipse detection method. This method first uses the arc-support line segment method to extract ellipse arc segments and then employs a hierarchical quadrant division strategy with a “coarse-to-fine” approach, integrating multiple constraints such as angle, quadrant, and relative position to combine arc segments and generate ellipse candidates. It uses a comprehensive score based on edge density, global coverage and local continuity to select the optimal ellipse from among the valid ellipses. Finally, a dynamic arc segment pruning method is introduced to dynamically remove relevant arcs from optimal ellipses, obtaining high-quality and non-redundant detection results. This method can achieve robust ellipse detection even when docking ring contours are partially obscured by shadows from robotic arms or nozzles.

## 1. Introduction

With the continuous advancement of aerospace technology, missions such as on-orbit service of failed spacecraft and space debris removal are becoming increasingly common [1,2,3]. Estimating the six degrees of freedom (6DoF) relative pose of non-cooperative spacecraft is fundamental to the success of these missions [4,5]. The key components of spacecraft, including satellite docking rings [6] and engine nozzles [7], project as ellipses onto two-dimensional images and can be utilized to obtain the spacecraft’s relative pose [8,9]. Recently, optical sensors like Time-of-Flight (ToF) cameras have also shown great potential in this domain. For example, methods based on circular features from ToF data have been developed for the pose estimation [10,11] and real-time tracking [12] of non-cooperative satellites, demonstrating the critical role of precise feature extraction. Therefore, the high-precision detection of ellipse targets directly impacts the accuracy and robustness of pose estimation. However, in complex scenarios such as when the docking ring is obscured by shadows from robotic arms or nozzles, the high-precision extraction of ellipse features faces many challenges, including severe contour loss, interference from redundant arc segments, and rapidly changing shadows. Therefore, developing an efficient and robust ellipse detection method is particularly important.

Existing elliptical detection methods can be mainly classified into three categories: methods based on Hough Transform (HT), methods based on edge-following, and methods based on deep learning.

Methods based on Hough Transform achieve ellipse detection by casting votes for arbitrary edge pixels into parameter space [13], but it has high computational complexity and large memory overhead. Therefore, more variants of the Hough Transform have been proposed. For instance, the Randomized Hough Transform [14,15] reduces computational load by randomly selecting edge points, but its accuracy remains insufficient in complex scenes.

Methods based on edge-following obtain continuous edge segments through edge detection [16], then fit ellipses using least squares [17] or RANSAC [18]. Additionally, optimization strategies specifically designed for short-arc ellipse fitting have been proposed to improve navigation precision [19], addressing the instability of fitting partial contours. Prasad et al. [20] segmented ellipse arcs based on curvature and concavity, and sorted edge arcs using the 2D Hough transform, but this approach incurs high computational time. Jia et al. [21] used characteristic numbers to reduce the number of arc combinations yet faced issues of missed detections and insufficient robustness. Lu [22] detected arc-support line segment method in grayscale images using LSD for ellipse detection. Meng et al. [23] proposed an Arc Adjacency Matrix-based Ellipse Detection (AAMED) method achieving rapid detection.

Methods based on deep learning use Convolutional Neural Networks (CNNs) to regress ellipse parameters, which allows for high-precision ellipse detection. Dong et al. proposed a CNN-based ellipse detector [24], which employs the U-NET structure to handle occlusion issues. However, this approach suffers from long training times and certain false detection rates. ElDet [25] developed an anchor-free general ellipse detection method that improves detection performance by combining and strengthening edge information in images. However, the robustness of this approach requires further improvement. Jia et al. [26] proposed a model-aware elliptical detection network (EDNet), which significantly improved detection accuracy for partially occluded ellipses. However, the method exhibits limitations when processing images containing concentric ellipses and lacks real-time capability.

To address the issues of low efficiency and insufficient robustness in the aforementioned methods for docking ring detection, this paper proposes an efficient and robust ellipse detection method. By integrating a hierarchical quadrant division strategy with a dynamic arc segment pruning mechanism, it effectively reduces candidate combinations and improves combination accuracy. Additionally, a comprehensive score is introduced to quantify ellipse fitting performance. The contributions of the proposed method are:

1. Hierarchical quadrant division strategy based on features. Proposes a “coarse-to-fine” quadrant division mechanism, prioritizing rapid four-quadrant division while performing eight-quadrant division only on residual valid arc segments. This reduces candidate combinations while preventing missed detections due to arc segment breaks.
2. Elliptical optimization mechanism based on dynamic arc segment pruning. An iterative pruning process based on arc segment-ellipse scores is designed. For each arc segment, the optimal ellipse candidate is retained while dynamically removing its redundant effective arc segments. This progressively yields a final detection set free of redundancy, significantly reducing duplicate detections and false positives.
3. Comprehensive score based on multi-dimensional feature constraints. A comprehensive score based on edge density, global coverage and local continuity is proposed for ellipse validation and screening, enhancing the accuracy and robustness of final detections.

The paper is structured as follows: Section 2 details the workflow of the proposed method; Section 3 presents the experiment results; Section 4 summarizes the paper and looks forward to future work.

## 2. The Proposed Method

**Figure 1:**
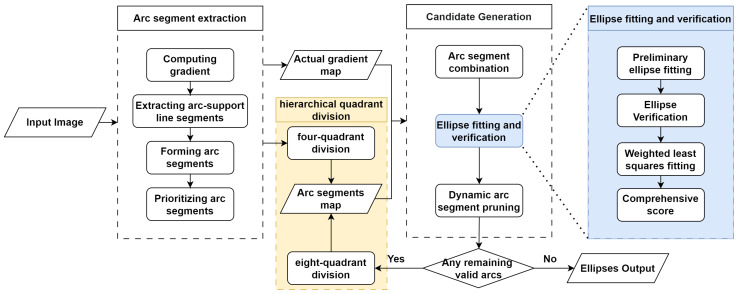
The workflow and examples of the proposed method.

This section details the workflow of the proposed method, as shown in Figure 1. Our proposed method can be divided into five parts: arc segment extraction, hierarchical quadrant division, arc segment combination, ellipse fitting and verification, and dynamic arc segment pruning.

### 2.1. Arc Segment Extraction

**Figure 2:**
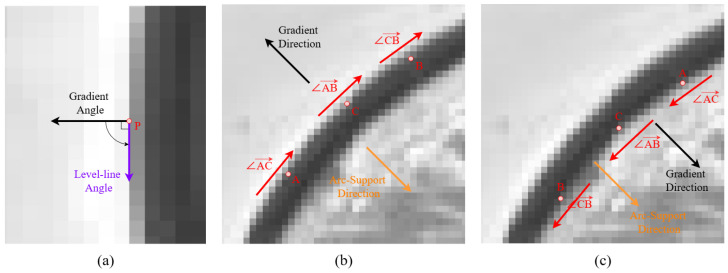
Diagram of arc-support line segment. (**a**) Level-line angle (rotated $90^{{}^{\circ}}$ clockwise from the gradient direction). (**b**) A case where the gradient direction aligns with the arc-support direction, and the angles change in a counter-clockwise manner. (**c**) Counter-example to (**b**).

During the ellipse fitting process, the first step involves extracting high-quality arc segments from the image. This paper adopts arc-support line segment method [27,28] to extract arc segments. Arc-support line segments are distributed along the curve, exhibit concave and convex features with sub-pixel precision, and point toward the ellipse center. First, the gradient magnitude and direction are computed for each pixel in the grayscale image. After removing noisy pixels, region growing aggregates pixels with similar gradient directions and spatial adjacency into support regions, yielding line segments along with their level-line angles (rotated $90^{{}^{\circ}}$ clockwise from the gradient direction). When the level-line angles within a segment’s region exhibit monotonic variation with the same rotation direction, it is labeled as an arc-support segment. Positive or negative polarity is assigned based on whether the rotation direction aligns with the gradient direction, as shown in Figure 2.

Subsequently, the algorithm analyzes the endpoint distances, principal direction angles, and polarity consistency of the arc-support line segments. It regards adjacent arc-support line segments with the same direction and polarity as potential connection candidates. Meanwhile, it counts the number of support points near each arc-support line segment’s endpoint and prioritizes connecting the segments with the most support points. It also eliminates straight-line segments and noise interference, ultimately forming ellipse arc segments. These extracted arc segments not only retain the convexity and polarity information but also have good spatial continuity, providing high-quality input for subsequent ellipse fitting. To facilitate implementation and ensure reproducibility, the complete workflow is formally summarized in Algorithm 1.
**Algorithm 1** Arc Segment Extraction**Require:** Input Image *I***Ensure:** Set of arc segments $S_{arc}$ 1: Compute gradient magnitude and direction for each pixel in *I* 2: Extract initial line segments $L_{init}$ using region growing 3: $L_{support}\leftarrow ⌀$ 4: **for all** $l\in L_{init}$ **do** 5:        **if** angles within *l*’s region exhibit monotonic variation **then** 6:            Label *l* as arc-support segment and determine polarity 7:            $L_{support}\leftarrow L_{support}\cup \left\{l\right\}$ 8:            Status[l]←Unused 9:     **end if**10: **end for**11:
$S_{arc}\leftarrow ⌀$12: **while** exists $l\in L_{support}$ with Status[l]=Unused **do**13:        Initialize current arc segment $A_{curr}\leftarrow \left\{l\right\}$14:        Status[l]←Used15:        Growth_Flag←True16:        **while** Growth_Flag is True **do**17:            Growth_Flag←False18:            Identify endpoints of current arc segment $A_{curr}$19:            Find set C of unused neighbor segments near endpoints20:            Filter C by constraints:21:               1. Same direction and polarity as $A_{curr}$22:               2. Satisfy distance and angle requirements23:            **if** C≠⌀ **then**24:               Select $l_{best}\in C$ with maximum support points count25:               Merge $l_{best}$ into $A_{curr}$26:               $Status[l_{best}]\leftarrow \mathrm{Used}$27:               Growth_Flag←True28:            **end if**29:        **end while**30:        $S_{arc}\leftarrow S_{arc}\cup \left\{A_{curr}\right\}$31: **end while**32: **return** $S_{arc}$

**Figure 3:**
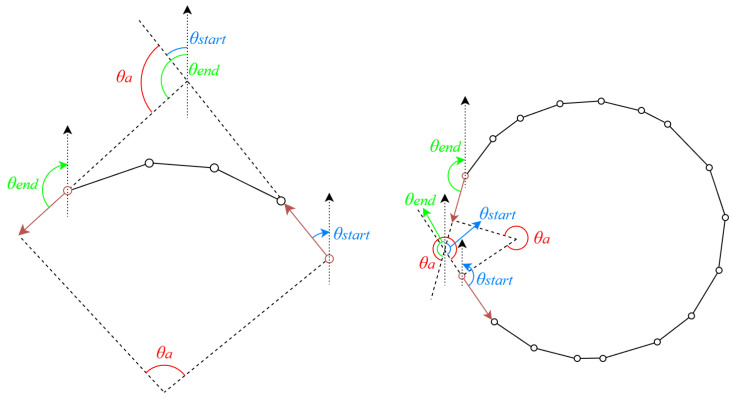
The coverage angle of the arc segment.

Before arc segment combination and ellipse fitting, we establish a prioritization mechanism to ensure that arc segments containing richer geometric information are processed first. We utilize two geometric features for sorting: the coverage angle $\theta_{a}$ and the pixel length. Arc segments are sorted by their coverage angle in descending order; if the angles are identical, they are subsequently sorted by pixel length. Specifically, since the arc segments are constructed from a sequence of arc-support line segments, $\theta_{a}$ represents the angular span determined by the orientation difference between the first and the last arc-support line segment. As shown in Figure 3, $\theta_{a}$ is calculated as the normalized angular difference between the orientation of the last segment ($\theta_{\mathrm{end}}$) and the first segment ($\theta_{\mathrm{start}}$):

(1) $$\theta_{a}=\left\{\begin{array}{cc} \theta_{\mathrm{end}}\;-\;\theta_{\mathrm{start}} & \mathrm{if}\;\theta_{\mathrm{end}}\geq \theta_{\mathrm{start}}\\ \theta_{\mathrm{end}}\;-\;\theta_{\mathrm{start}}\;+\;360^{{}^{\circ}} & \mathrm{if}\;\theta_{\mathrm{end}}<\theta_{\mathrm{start}} \end{array}\right.$$

where $\theta_{a}\in \left(0,360^{{}^{\circ}}\right]$, and both $\theta_{\mathrm{end}}$ and $\theta_{\mathrm{start}}$ are defined in range $\left[-180^{{}^{\circ}},180^{{}^{\circ}}\right]$.

### 2.2. Hierarchical Quadrant Division

Traditional ellipse detection algorithms typically classify arc segments into fixed quadrants and require complementary quadrant distributions during fitting. This strategy is simple and efficient, performing well when the arc segments are complete and unobstructed. However, when images contain local occlusions or noise, continuous arc segments originally belonging to the same ellipse may break into multiple segments, and these fragmented arc segments may contain the same quadrants in their distribution. At this point, the quadrant complementarity strategy under the four-quadrant division will make it difficult to combine these fragmented arc segments, leading to missed detections. Although subdividing the angle space into eight quadrants or allowing same-quadrant pairing can solve this problem, it significantly increases candidate combinations and computational overhead, compromising method efficiency and real-time performance.

To address this issue, this paper proposes a hierarchical quadrant division strategy that follows a “coarse-to-fine” approach: in the initial stage, a four-quadrant division is used to rapidly combine invalid arc segments; subsequently refining only the residual arc segments that remained unpaired during the initial stage and possess sufficient significance are refined into eight quadrants, followed by arc segment combination and ellipse fitting. The specific implementation is as follows:

First, the angle space $\left[0,360^{{}^{\circ}}\right)$ is evenly divided into four quadrants.

(2) $$Q_{i}=\left[\left(i-1\right)\times 90^{{}^{\circ}},\;i\times 90^{{}^{\circ}}\right),\;i\in \left\{1,2,3,4\right\}.$$

For each arc segment, sequentially traverse all its arc-support line segments, calculate the gradient direction $\theta_{g,l}$ of each line segment and map it to the corresponding quadrant, and then generate a 4-bit quadrant mask accordingly, as shown in the formula.

(3) $$mask^{4}=\left(b_{4},b_{3},b_{2},b_{1}\right),\;b_{i}=\left\{\begin{array}{cc} 1, & \exists \;\theta_{g,l}\in Q_{i}.\\ 0, & \mathrm{otherwise}. \end{array}\right.$$

Here, the i-th bit being 1 indicates that the arc segment covers the i-th quadrant. If an arc segment spans multiple quadrants, all corresponding bits are set to 1. For instance, $mask^{4}=0110$ indicates the arc segment spans the second and third quadrants; similarly, $mask^{4}=0111$ represents an arc spanning the first, second, and third quadrants.

This quadrant mask representation significantly affects the algorithm’s execution efficiency by acting as a rapid filter during the arc segment combination phase. By performing a bitwise AND operation on the masks of two arc segments, we can determine if they overlap in the angular space. If $mask_{1}^{4}\;\&\;mask_{2}^{4}\neq 0$, the two arc segments occupy at least one common quadrant, indicating a conflict. Such combinations are immediately discarded without performing ellipse fitting.

In the initial stage, $mask^{4}$ is used to rapidly combine arc segments and perform ellipse fitting while eliminating invalid arc segments. If there are still two or more uncombined arc segments with large coverage angles after the initial stage, an 8-bit quadrant mask refinement is applied to the remaining arc segments, followed by another round of arc segment combination and ellipse fitting.

Our strategy efficiently generates candidate ellipses in most scenarios. In locally complex or heavily occluded cases, quadrant refinement prevents missed detections, balancing efficiency and completeness.

### 2.3. Arc Segment Combination

To generate high-quality candidate ellipses, this paper proposes an arc segment combination strategy based on significance and multiple constraints. The overall approach is as follows: if the coverage angle $\theta_{a}$ of an arc segment exceeds the individual fitting threshold $\theta_{a}^{\mathrm{th}}$, it is classified as a significant arc segment and allowed to be used alone for ellipse fitting; then, following the sorted order, arc segments are sequentially selected and combined in pairs to fit ellipses. Specifically, for a current arc segment, we will sequentially search the remaining list for potential partners. To improve the effectiveness of pairing and computational efficiency, we introduce three types of constraints to eliminate invalid arc segment combinations.

**Figure 4:**
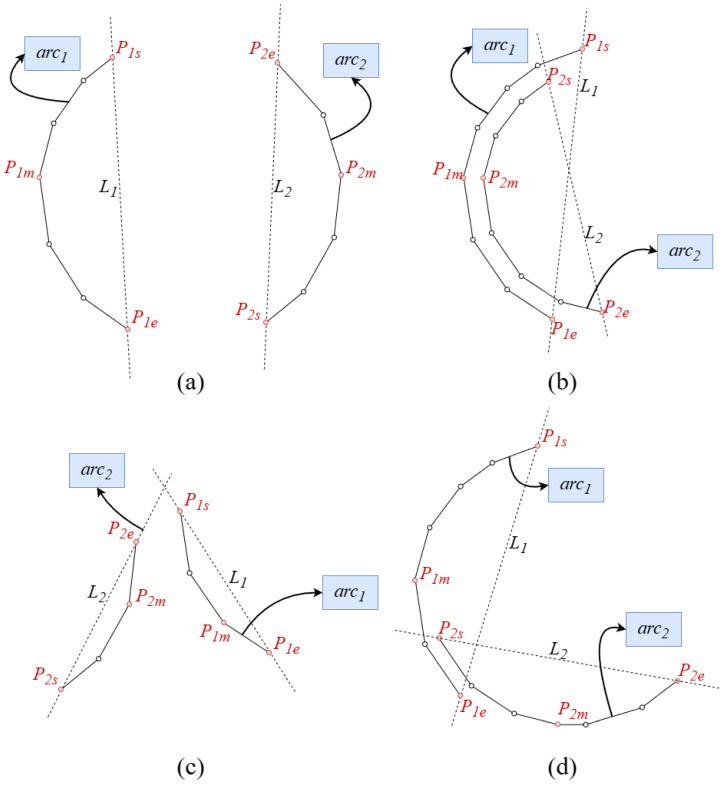
Relative position constraint. (**a**) is a situation that satisfies the relative position constraint. (**b**–**d**) are situations that do not satisfy the relative position constraint.

1. Angle Constraint: The sum of the coverage angles of the two arc segments must exceed the combined angle threshold $\theta_{\mathrm{sum}}^{\mathrm{th}}$. This constraint effectively eliminates combinations of arc segments with insufficient coverage angles, preventing the generation of numerous erroneous ellipses.
2. Quadrant Constraint: To ensure that the arc segment combinations have complementary quadrant distributions, no overlapping quadrants may exist between two segments; that is, the quadrant masks of the two arc segments have no intersection in bits. For two quadrant masks $mask_{1}^{k}$ and $mask_{2}^{k}$, where k∈{4,8}, if $mask_{1}^{k}\;\&\;mask_{2}^{k}=0$, then no overlapping quadrants exist between the two arc segments. This constraint can be applied using either a four-quadrant or eight-quadrant mask.
3. Relative Position Constraint: To avoid combining geometrically unreasonable arc segments, a directed distance determination based on endpoints and arc interior points is introduced. Let the endpoints of the arc segment $arc_{1}$ be ($P_{1s}$, $P_{1e}$), and the endpoints of the arc segment $arc_{2}$ be ($P_{2s}$, $P_{2e}$). Arbitrarily select interior points as reference points $P_{1m}\in arc_{1}$, $P_{2m}\in arc_{2}$. Let line $L_{1}$ connect $P_{1s}$ and $P_{1e}$, and line $L_{2}$ connect $P_{2s}$ and $P_{2e}$. We define a function δ(P,L) to represent the directed distance from a point *P* to a line *L*. Unlike standard Euclidean distance, the directed distance carries a positive or negative sign depending on which side of the line the point is located. Consequently, the condition that the product of two distances is negative implies that the distances have opposite signs. Geometrically, this ensures that the reference points lie on opposite sides of the connecting line. Valid arc combinations must satisfy the following condition:
  (4) $$\left\{\begin{array}{c} \delta \left(P_{1m},L_{1}\right)\;\times \;\delta \left(P_{2m},L_{1}\right)<0\\ \delta \left(P_{1m},L_{2}\right)\;\times \;\delta \left(P_{2m},L_{2}\right)<0 \end{array}\right.$$
  Furthermore, to further exclude cases that satisfy the above constraints but do not constitute valid arc segment combinations, such as case c in Figure 4, the following condition must also be met:
  (5) $$\left\{\begin{array}{c} \delta \left(P_{2s},L_{1}\right)\;\times \;\delta \left(P_{2e},L_{1}\right)>0\\ \delta \left(P_{1s},L_{2}\right)\;\times \;\delta \left(P_{1e},L_{2}\right)>0 \end{array}\right.$$

### 2.4. Ellipse Fitting and Verification

#### 2.4.1. Preliminary Ellipse Fitting

The standard equation of an ellipse is expressed as:

(6) $$Ax^{2}+Bxy+Cy^{2}+Dx+Ey+F=0$$

For significant arc segments or constrained candidate arc segment combinations, initial parameters {A,B,C,D,E,F} are obtained by direct least squares fitting [29]. This method has a low computational cost and can quickly fit the candidate arc segment combinations to obtain an initial model for subsequent geometric verification.

#### 2.4.2. Ellipse Verification

Based on the ellipse model obtained from the preliminary fitting, we need to identify valid edge points from the image edges to determine the accuracy of the ellipse. Valid edge points must satisfy two conditions: First, their shortest distance d(p,e) to the ellipse boundary must meet the pixel threshold $d\left(p,e\right)\leq T_{\mathrm{pix}}$. Here, d(p,e) is defined as the shortest Euclidean distance from an edge point *p* to the nearest point on the contour of the fitted ellipse *e*. Second, we define $\tau_{\theta }$ as the gradient orientation consistency threshold, their actual gradient direction $\theta_{\mathrm{obs}}\left(p\right)$ must meet the threshold $|\theta_{\mathrm{obs}}\left(p\right)-\theta_{\mathrm{theo}}\left(x,y\right)|<\tau_{\theta }$ relative to the theoretical gradient direction $\theta_{\mathrm{theo}}\left(x,y\right)$. Here, $\theta_{\mathrm{obs}}\left(p\right)$ is calculated by the Canny operator and $\theta_{\mathrm{theo}}\left(x,y\right)$ is calculated based on the point’s coordinates and the ellipse parameters.

(7) $$\theta_{\mathrm{theo}}\left(x,y\right)=atan2\left(B\;x+2C\;y+E,\;2Ax+B\;y+D\right)$$

The validity determination process for a single arc segment is as follows:

1. Iterate through each arc-support line segment of the arc segment, and search for valid edge points within a neighborhood defined by the distance threshold $T_{\mathrm{pix}}$ around each arc-support line segment. To suppress noise, limit the maximum number of valid edge points counted within this neighborhood to the length of the arc-support line segment.
2. Count the total number of valid edge points along the specific arc segment being verified. We calculate the ratio of valid points to the total pixel length of this specific arc segment. We define an edge validity threshold, denoted as $T_{\mathrm{ratio}}$. If this ratio exceeds $T_{\mathrm{ratio}}$, the arc segment is verified as a true part of the ellipse and retained; otherwise, it is regarded as a noise segment. This threshold applies to the local quality of arc segments, not the global completeness of the ellipse.

When all arc segments participating in the fitting are verified as valid, we expand the search to find other arc segments near the candidate ellipse. We perform the same validity judgment on these arc segments and summarize the total number of valid edge points, denoted as $N_{\mathrm{edge}}$. To rapidly assess the coverage of edge points on the ellipse, we map all valid edge points’ polar angles on the candidate ellipse to a discrete angular space. Specifically, the polar angles are calculated relative to the geometric center $\left(x_{c},y_{c}\right)$ of the candidate ellipse corresponding to the valid edge points. The angular space is discretized into 360 bins of $1^{{}^{\circ}}$ each. A bin is considered ‘occupied’ if it contains valid edge points. Based on this, the total coverage angle $\Theta_{\mathrm{cov}}\left(S\right)$ is defined as the angular difference between the maximum and minimum occupied bins. Furthermore, the maximum continuous coverage angle $\Theta_{\max}$ is defined as the angular span of the longest unbroken sequence of consecutively occupied bins within this angular space.

A candidate ellipse is deemed valid only if it satisfies two simultaneous conditions. First, the ratio of the total number of edge points $N_{\mathrm{edge}}$, to the ellipse circumference $L_{\mathrm{ell}}$, must exceed the edge point density threshold $T_{\mathrm{den}}$. Second, the coverage angle of valid edge points $\Theta_{\mathrm{cov}}\left(S\right)$, must surpass the total coverage angle threshold $T_{\mathrm{ang}}$. Valid edge points are then used for precise fitting. The circumference $L_{\mathrm{ell}}$ of the ellipse is primarily calculated using Ramanujan’s approximate perimeter formula, where *a* represents the major axis and *b* the minor axis of the ellipse. Specifically, the parameters for the major and minor axes of the ellipse are derived mathematically from the initial parameters of the ellipse equation obtained in Section 2.4.1.

(8) $$L_{\mathrm{ell}}\approx \pi \left[3\left(a+b\right)-\sqrt{\left(3a+b)(a+3b\right)}\right]$$

#### 2.4.3. Weighted Least Squares Ellipse Fitting

In actual images, the reliability of different edge points varies. Points with higher gradient magnitudes and gradient directions highly consistent with the ellipse model are typically more representative. To suppress noise and interference from non-elliptical structures, this paper employs weighted least squares for precise ellipse fitting [30,31].

For an effective edge point, let its normalized gradient magnitude be $G_{i}$, and let the normalized difference between its actual gradient direction and theoretical gradient direction be $\Delta \phi_{i}$. Then, the weight $w_{i}$ can be defined as:

(9) $$\begin{array}{cc} w_{i} & =2G_{i}\cos\left(\Delta \phi_{i}\right)=2∥\nabla I\left(x_{i},y_{i}\right)∥\cos\;\left(\left|\frac{\theta_{\mathrm{obs}}\left(p\right)-\theta_{\mathrm{theo}}\left(x,y\right)}{\tau_{\theta }}\right|\right) \end{array}$$

At this point, the ellipse fitting problem can be formulated as minimizing the weighted mean square error. The resulting ellipse parameters constitute a more precise ellipse model, thereby enhancing the accuracy of the candidate ellipse.

(10) $$\min\;\sum_{i=1}^{n}w_{i}(Ax_{i}^{2}+Bx_{i}y_{i}+Cy_{i}^{2}+Dx_{i}+Ey_{i}{+F)}^{2}$$

This problem is equivalent to minimizing the quadratic form $u^{T}Su$ subject to a normalization constraint (e.g., ∥u∥=1), where $u={\left[A,B,C,D,E,F\right]}^{T}$ is the parameter vector.

S is the 6×6 weighted scatter matrix, constructed as:

(11) $$S=\sum_{i=1}^{n}w_{i}v_{i}v_{i}^{T}$$

where $v_{i}={\left[x_{i}^{2},x_{i}y_{i},y_{i}^{2},x_{i},y_{i},1\right]}^{T}$ is the design vector for the *i*-th edge point, with $\left(x_{i},y_{i}\right)$ denoting its position coordinates in the image.

To obtain the optimal parameters, we perform Eigenvalue Decomposition (EVD) on the matrix S. The solution u is the eigenvector corresponding to the smallest non-negative eigenvalue. In our implementation, this is numerically solved using the QR algorithm.

#### 2.4.4. Comprehensive Score

To strictly evaluate the quality of the detected ellipses, this paper proposes an effective comprehensive ellipse score, integrating the total number of edge points $N_{\mathrm{edge}}$ and the angular metrics calculated in Section 2.4.2:

(12) $$\mathrm{Score}=\alpha_{1}\times \frac{N_{\mathrm{edge}}}{L_{\mathrm{ell}}}+\alpha_{2}\times \frac{\Theta_{\mathrm{cov}}\left(S\right)}{2\times \pi }+\alpha_{3}\times \frac{\Theta_{\max}}{\Theta_{\mathrm{cov}}\left(S\right)}$$

Here, $N_{\mathrm{edge}}$ denotes the total number of edge points falling on the candidate ellipse, $L_{\mathrm{ell}}$ represents the ellipse’s theoretical circumference, and *S* is the set of valid edge points. $\Theta_{\mathrm{cov}}\left(S\right)$ is the total coverage angle of valid edge points on the candidate ellipse, while $\Theta_{\max}$ is the maximum continuous coverage angle. These angular metrics are determined relative to the fitted ellipse center as defined in Section 2.4.2. The weight coefficients $\alpha_{1}$, $\alpha_{2}$, and $\alpha_{3}$ balance the importance of edge density, global coverage, and local continuity.

This score reflects edge point density, overall coverage of edge points, and local continuity of edge points. The comprehensive score enables our algorithm to prioritize optimal ellipse models featuring a large number of valid edge points, continuous long arc segments, and evenly distributed valid edge points.

### 2.5. Dynamic Arc Segment Pruning

In fact, each arc segment should ultimately belong to only one detected ellipse. To prevent multiple combinations of the same ellipse’s arc segments, thereby reducing redundant detection and computational overhead, this paper proposes an iterative dynamic arc segment pruning strategy. The core idea is as follows: For each arc segment $Arc_{i}$ to be processed, search within the “pool of available arc segments” for another arc segment $Arc_{j}$ that can be paired with it to form an ellipse. Select the optimal ellipse from among these and remove all valid arc segments associated with that ellipse, continuing this process until all arc segments have been processed. The pseudo-code is presented in Algorithm 2.
**Algorithm 2** Ellipse Detection Based on Dynamic Arc Segment Pruning**Require:**  initial set of Arc segments A**Ensure:**  detected ellipses E 1: available_Arcs ←A 2:
E←⌀ 3: **while** available_Arcs ≠⌀ **do** 4:      **for all** ${\mathrm{Arc}}_{i}\in$ available_Arcs **do** 5:             best_score_*i*_ ←−∞ 6:             best_ellipse_*i*_ ← null 7:             **for all** ${\mathrm{Arc}}_{j}\in \;\mathrm{available}\_\mathrm{Arcs}\;\setminus \left\{{\mathrm{Arc}}_{i}\right\}$ **do** 8:                  **if** ConstraintsMet(${\mathrm{Arc}}_{i},{\mathrm{Arc}}_{j}$) **then** 9:                   $E_{ij}\leftarrow \mathrm{FitEllipse}\left({\mathrm{Arc}}_{i},{\mathrm{Arc}}_{j}\right)$10:                  ${\mathrm{score}}_{ij}\leftarrow \mathrm{ValidateEllipse}\left(E_{ij}\right)$11:                  **if** ${\mathrm{score}}_{ij}>{\mathrm{best}\_\mathrm{score}}_{i}$ **then**12:                       ${\mathrm{best}\_\mathrm{score}}_{i}\leftarrow {\mathrm{score}}_{ij}$13:                       ${\mathrm{best}\_\mathrm{ellipse}}_{i}\leftarrow E_{ij}$14:                  **end if**15:              **end if**16:          **end for**17:          **if** ${\mathrm{best}\_\mathrm{ellipse}}_{i}\neq \mathrm{null}$ **and** ${\mathrm{best}\_\mathrm{score}}_{i}>0$ **then**18:              RemoveArcs(best_ellipse_*i*_, available_Arcs)19:              $E\leftarrow E\cup \{{\mathrm{best}\_\mathrm{ellipse}}_{i}\}$20:          **end if**21:      **end for**22: **end while**23: **return** E

### 2.6. Summary

In summary, classical ellipse detection algorithms typically face a trade-off between robustness and efficiency. Methods based on Hough Transform offer robustness but incur high computational costs due to pixel-wise voting. Conversely, methods based on edge-following are efficient but highly sensitive to edge fragmentation and local occlusion.

The proposed algorithm effectively bridges this gap.

1. Unlike methods based on Hough Transform, our algorithm significantly reduces the candidate combinations by employing a hierarchical quadrant division strategy and dynamic arc segment pruning. These mechanisms prioritize high-quality arc segments and iteratively remove redundancies, ensuring real-time performance.
2. Unlike methods based on edge-following, our method utilizes a hierarchical quadrant division strategy and a comprehensive score based on edge density, global coverage and local continuity. This allows the algorithm to robustly identify ellipses even when they are partially occluded or discontinuous, effectively achieving a balance between efficiency and robustness.

## 3. Experimental Results

To validate the algorithm’s performance, we evaluated it on five existing real-world datasets and real docking ring images.

To systematically evaluate the robustness of the proposed method, we define “Complex Scenes” as environments characterized by three challenging factors: noise, uneven illumination, and local occlusion.

1. Noise and illumination: Uneven illumination often leads to edge fragmentation, while background noise introduces false positives. The robustness against noise and uneven illumination is primarily evaluated in Section 3.4 using the five public datasets.
2. Occlusion: Local occlusion interrupts the continuity of the ellipse contour, resulting in fewer visible arc segments. The algorithm’s ability to handle local occlusion is specifically validated in Section 3.5 using the real docking ring images.

All experiments were conducted on a computer with 16G RAM and an Intel i7-8700 3.20 GHz CPU (Intel, Santa Clara, CA, USA).

### 3.1. Dataset

We used five real-world datasets, including two satellite datasets, the Smartphone dataset, the PCB dataset, and the Prasad dataset. Satellite 1 dataset [23] consists of satellite images captured by the OEDMS camera of the NextSat spacecraft, with a resolution of 318 × 318. All images are affected by illumination and noise. Satellite 2 dataset [23] consists of satellite images captured by the infrared camera of the NextSat spacecraft, with a resolution of 239 × 317. Besides being affected by illumination and noise, these images are also influenced by object texture. The Smartphone dataset [32] is derived from videos captured by a smartphone, with a resolution of 640 × 480. It contains images of traffic signs and bicycles, presenting challenges such as noise and blurring. The PCB dataset [27] is composed of industrial PCB images with a resolution of 512 × 456. Most of them are concentric circles and are subject to a large amount of noise. The Prasad dataset [20] consists of real images of varying resolutions, containing ellipses of different sizes and quantities, and the image backgrounds are relatively complex.

**Figure 5:**
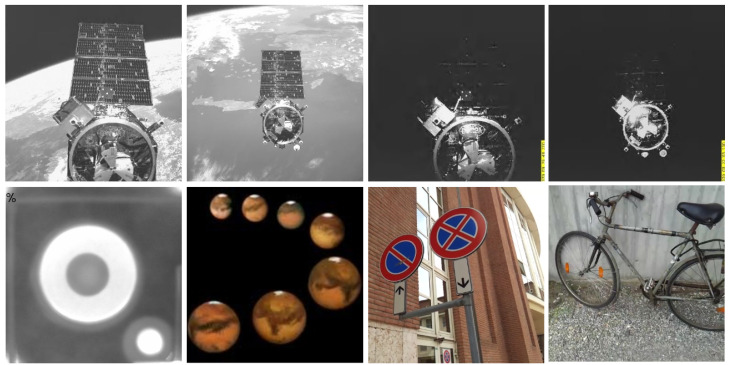
Five sample images from the datasets.

Examples from the five real-world datasets are shown in Figure 5.

### 3.2. Evaluation Metrics

To compare with other methods, this paper uses accuracy, recall, F-measure, and detection time as performance evaluation metrics. The definitions of accuracy *P*, recall *R*, and F-measure *F* are given in [20,33] as follows:

(13) $$P=\frac{\mathrm{Number}\;\mathrm{of}\;\mathrm{correctly}\;\mathrm{detected}\;\mathrm{ellipses}}{\mathrm{Total}\;\mathrm{number}\;\mathrm{of}\;\mathrm{ellipses}\;\mathrm{detected}}$$

(14) $$R=\frac{\mathrm{Number}\;\mathrm{of}\;\mathrm{correctly}\;\mathrm{detected}\;\mathrm{ellipses}}{\mathrm{Total}\;\mathrm{number}\;\mathrm{of}\;\mathrm{ellipses}\;\mathrm{present}\;\mathrm{in}\;\mathrm{test}\;\mathrm{images}}$$

(15) $$F=\frac{2}{P^{-1}+R^{-1}}$$

The correctness of an ellipse is determined by the overlap ratio *M* between the detected ellipse and the true ellipse [20], which is calculated as follows, where $E_{d}$ represents the detected ellipse and $E_{g}$ represents the true ellipse. If *M* exceeds 0.8, the detected ellipse is considered a correct ellipse.

(16) $$M\left(E_{d},E_{g}\right)=\frac{\mathrm{area}(E_{d}\cap E_{g})}{\mathrm{area}(E_{d}\cup E_{g})}$$

### 3.3. Parameter Analysis and Visualization

To facilitate reproducibility and intuitively illustrate the algorithmic process, this section provides a detailed definition and sensitivity analysis of the key thresholds and weights, followed by a step-by-step visualization of the detection results. The parameter values, summarized in Table 1, were empirically selected to strike a robust balance among detection accuracy, recall, and computational efficiency. The specific rationale and trade-off analysis for each key parameter are detailed below.

#### 3.3.1. Individual Fitting Threshold

The individual fitting threshold $\theta_{a}^{\mathrm{th}}$ is set to $120^{{}^{\circ}}$ to determine whether an arc segment possesses sufficient angular span for direct fitting. The selection of this value represents a critical trade-off between detection recall and computational stability. Specifically, setting $\theta_{a}^{\mathrm{th}}$ too high (e.g., $150^{{}^{\circ}}$) poses a risk to isolated arcs; valid segments spanning $140^{{}^{\circ}}$, which are geometrically distinct enough to define an ellipse, would be rejected, leading to a lower recall rate. Conversely, decreasing the threshold allows segments with limited angular span to be fitted individually. Even with sufficient pixel length, a narrow angular coverage implies insufficient curvature variation to uniquely constrain an ellipse model, making the fitting mathematically unstable and generating numerous redundant candidates. Consequently, $\theta_{a}^{\mathrm{th}}=120^{{}^{\circ}}$ is empirically identified as the optimal balance to capture significant single-arc ellipses while maintaining efficiency.

#### 3.3.2. Combined Angle Threshold

The combined angle threshold $\theta_{\mathrm{sum}}^{\mathrm{th}}$ serves as a prerequisite for arc segment combination and is set to $80^{{}^{\circ}}$ to ensure that a pair of arc segments provides sufficient angular span to constrain the degrees of freedom of an ellipse equation. Specifically, increasing $\theta_{\mathrm{sum}}^{\mathrm{th}}$ (e.g., >$100^{{}^{\circ}}$) imposes strict constraints that are detrimental in non-cooperative docking scenarios, where target rings are often partially occluded by robotic arms or shadows. Under such conditions, contours break into fragmented arcs; a high threshold would filter out valid combinations of these fragments, thereby significantly decreasing the recall rate. Conversely, decreasing $\theta_{\mathrm{sum}}^{\mathrm{th}}$ (e.g., <$60^{{}^{\circ}}$) permits the pairing of arc segments with insufficient coverage angle. From the perspective of least squares fitting, deriving an ellipse from a narrow angular range can result in unstable parameters where slight noise results in large shape deviations. Moreover, a lower threshold triggers an increase in the number of arc segment combinations, increasing computational cost without improving detection accuracy. Therefore, $\theta_{\mathrm{sum}}^{\mathrm{th}}=80^{{}^{\circ}}$ ensures both the reliability of the algorithm and the maintenance of computational efficiency.

#### 3.3.3. Pixel Distance Threshold

The distance threshold $T_{\mathrm{pix}}$, used to validate edge points against the fitted ellipse, is set to 3 pixels. This choice reflects a necessary balance between accommodating inherent fitting residuals and suppressing noise. Specifically, increasing $T_{\mathrm{pix}}$ expands the search neighborhood, which leads to two detrimental effects: it significantly raises the computational cost by requiring the verification of more pixels, and it increases the likelihood of incorporating noise or irrelevant edge structures into the model. Conversely, reducing $T_{\mathrm{pix}}$ (e.g., to 1 or 2 pixels) imposes an overly restrictive constraint. Given that candidate ellipses are generated via least-squares fitting, slight spatial deviations between the fitted curve and actual edge pixels are inevitable due to discrete image quantization. A threshold lower than 3 pixels is often insufficient to tolerate these expected deviations, resulting in the erroneous rejection of valid edge points. Consequently, $T_{\mathrm{pix}}=3$ is empirically selected to accommodate fitting errors while ensuring computational efficiency.

#### 3.3.4. Gradient Orientation Consistency Threshold

The gradient orientation consistency threshold $\tau_{\theta }$ is set to $10^{{}^{\circ}}$ to verify whether the actual gradient direction of an edge pixel aligns with its theoretical gradient direction on the candidate ellipse. This parameter represents a necessary compromise between tolerance for environmental variations and geometric precision. Specifically, reducing $\tau_{\theta }$ (e.g., <$5^{{}^{\circ}}$) assumes idealized imaging conditions; however, in real-world spacecraft imagery, local illumination changes and sensor noise inevitably cause the observed gradient directions to deviate from the theoretical model. An excessively narrow threshold fails to account for these physical deviations, causing valid edge points to be rejected and leading to a significant drop in the recall rate. Conversely, increasing $\tau_{\theta }$ (e.g., >$20^{{}^{\circ}}$) relaxes the consistency constraint, allowing spurious edge pixels or background noise to be incorrectly associated with the ellipse candidate. This would compromise the accuracy of the validity assessment—potentially leading to misjudgments—and unnecessarily increase computational overhead due to the algorithm processing irrelevant data. Consequently, $\tau_{\theta }=10^{{}^{\circ}}$ based on experience ensures precise edge association.

#### 3.3.5. Edge Validity Threshold

The threshold $T_{\mathrm{ratio}}$ serves as the criterion for determining the validity of an arc segment based on its edge support ratio, set to 80%. This selection reflects a critical balance between noise tolerance and verification strictness. Specifically, setting $T_{\mathrm{ratio}}$ to an excessively high value (e.g., 90%) imposes an overly stringent constraint. In real-world images, even genuine arc segments are prone to minor pixel-level discontinuities or noise; a strict threshold would cause these valid but imperfect segments to be discarded, thereby diminishing the recall rate. Conversely, decreasing $T_{\mathrm{ratio}}$ (e.g., to 0.5) relaxes the validity criterion, allowing spurious or noisy segments to be misclassified as valid. Consequently, incorrect candidate ellipses accumulate an inflated number of valid edge points, causing them to erroneously pass the verification stage. This not only leads to false positives but also increases the computational burden for subsequent steps. Consequently, $T_{\mathrm{ratio}}=0.8$ is empirically identified as the optimal threshold to reliably identify valid arc segments with noise and discontinuities while maintaining verification robustness.

#### 3.3.6. Edge Point Density Threshold and Total Coverage Angle Threshold

The edge point density threshold $T_{\mathrm{den}}$ and the total coverage angle threshold $T_{\mathrm{ang}}$ are jointly set to 0.4 and $150^{{}^{\circ}}$. These settings represent a trade-off between occlusion robustness and false-positive suppression. Specifically, elevating these thresholds (e.g., $T_{\mathrm{den}}>0.6$ or $T_{\mathrm{ang}}>200^{{}^{\circ}}$) imposes criteria that are unrealistic for non-cooperative docking missions. In such scenarios, the target ring is frequently obscured by robotic arms or structural shadows; excessively strict requirements would preemptively reject these valid but partially occluded ellipses, thereby significantly reducing the recall rate. Conversely, lowering these thresholds (e.g., $T_{\mathrm{den}}<0.2$ or $T_{\mathrm{ang}}<90^{{}^{\circ}}$) permits the admission of low-quality candidates. Allowing ellipses with few valid edge points or minimal angular span to enter the scoring stage has two adverse consequences: it unnecessarily increases the computational burden, and it compromises the discriminatory power of the comprehensive score, leading to a rise in false positives and a decrease in detection precision. Consequently, the values of $T_{\mathrm{den}}=0.4$ and $T_{\mathrm{ang}}=150^{{}^{\circ}}$ are empirically selected to preserve accuracy even under occlusion.

#### 3.3.7. Scoring Weights

In Equation (12), the weights $\alpha_{1},\alpha_{2},$ and $\alpha_{3}$ are universally set to 1. This uniform distribution balances global integrity with local continuity. Specifically, the metrics of edge density ($N_{\mathrm{edge}}/L_{\mathrm{ell}}$) and global coverage ($\Theta_{\mathrm{cov}}$) serve as the most robust indicators for ellipses, dominating the score in ideal conditions. However, in scenarios involving partial occlusion, these values naturally diminish. Under such conditions, the remaining visible segments usually retain high local continuity ($\Theta_{\max}/\Theta_{\mathrm{cov}}$), distinguishing them from random noise. Consequently, this equal weighting strategy ensures that the algorithm remains sensitive to both high-quality, complete ellipses and partially occluded targets that exhibit strong local continuity.

#### 3.3.8. Step-by-Step Visualization

**Figure 6:**
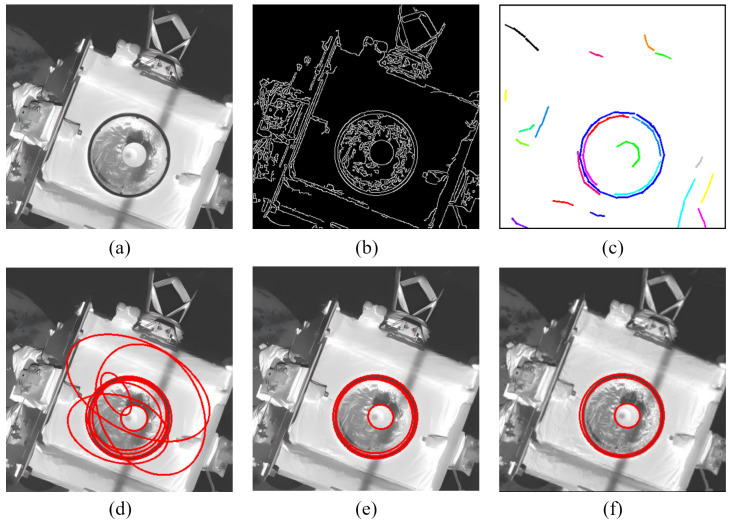
Overall detection example: (**a**) Original image. (**b**) Actual gradient map. (**c**) Arc segments, where different colors distinguish different arc segments (**d**) Fitted ellipse candidates. (**e**) Valid ellipses after verification. (**f**) Optimal ellipse.

Finally, to intuitively illustrate the functionality of each module in the proposed framework and verify the effectiveness of the intermediate steps, Figure 6 displays the partial results of the detection process on a real docking ring image.

The specific workflow visualized in Figure 6 proceeds as follows: First, (a) presents the original image. In the arc segment extraction phase, the actual gradient map shown in (b) is generated, followed by the arc segments map in (c) obtained using the arc-support line segment method. Next, (d) displays all fitted results from the arc segment combination and preliminary ellipse fitting phase after applying the dynamic arc segment pruning strategy. These candidates are further filtered by the ellipse verification module; (e) depicts the valid ellipses that satisfy the specific edge point density threshold and total coverage angle threshold. Finally, (f) presents the optimal ellipse set identified from these valid ellipses based on the comprehensive score.

### 3.4. Compare with Existing Methods

This paper compares four popular ellipse detection methods in recent years, including the Fornaciari method [32], Jia method [21], Lu method [22], and AAMED method [23].

**Figure 7:**
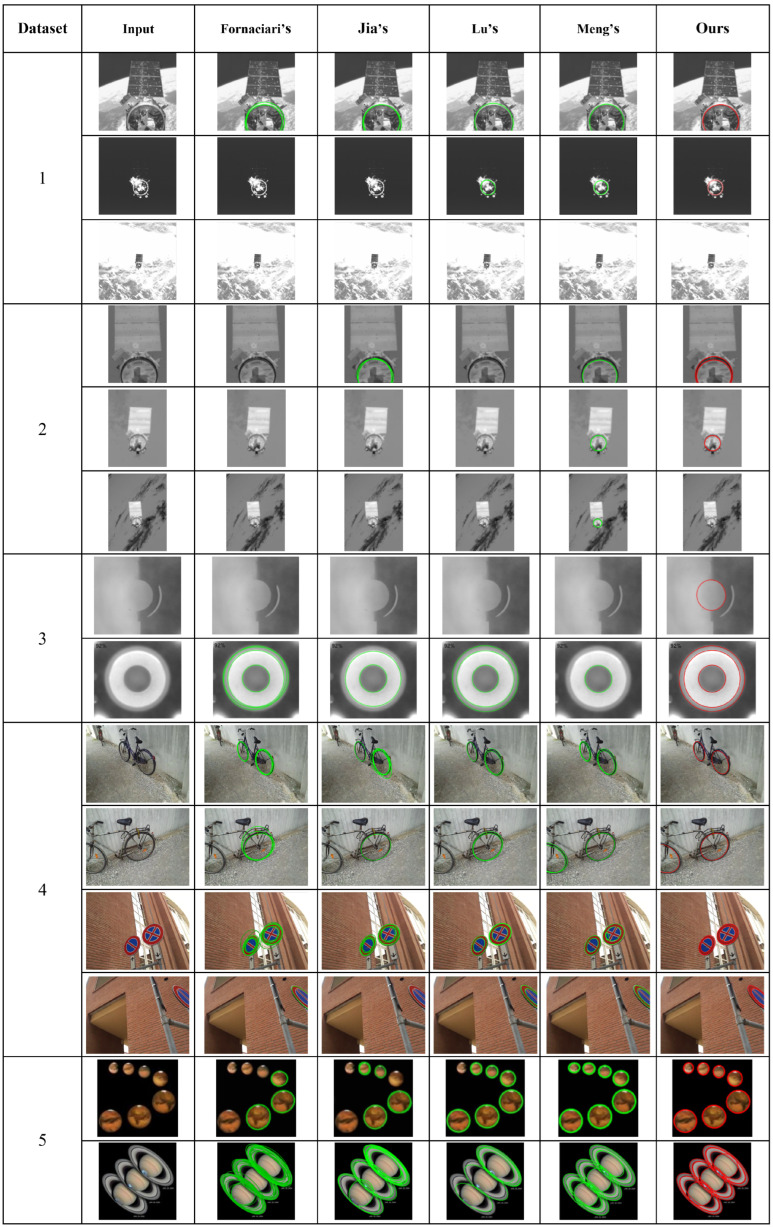
Five examples of ellipse detection on datasets, 1–5 being Satellite 1 dataset, Satellite 2 dataset, Smartphone dataset, PCB dataset, and Prasad dataset, respectively. The red ellipses indicate the detection results of our proposed algorithm, while the green ellipses represent the results of the comparison methods (Fornaciari’s [32], Jia’s [21], Lu’s [22], and Meng’s [23]).

The detection results on real datasets are shown in Table 2. Our algorithm achieves the highest accuracy through weighted least squares ellipse fitting and strictly screens the correct ellipses using a score. Therefore, our algorithm has a higher recall rate and F-measure than the Fornaciari, Jia, and Lu methods, achieving significant performance improvements. However, in the satellite dataset and Prasad dataset, the recall rate is not higher than that of the Meng algorithm, and the F-measure is not the best. The main reason is that when the pixel size of the ellipse is small in these datasets, it is difficult to generate enough arc segments for fitting the ellipse, and our algorithm detects fewer ellipses. Examples of ellipse detection in the five datasets are shown in Figure 7. The average algorithm time is shown in Table 3.

In spacecraft docking missions, enhancing the accuracy of ellipse detection is crucial for ensuring mission success. To improve detection precision, our proposed algorithm employs the arc-support line segment method to extract arc segments. However, this approach also increases the detection time, as evidenced by our detection times exceeding those of Fornaciari, Jia, and Meng across all five datasets. However, thanks to our multi-constraint combination and dynamic arc segment pruning strategy, our method eliminates the time-consuming ellipse clustering and ellipse verification steps in the Lu method, thus reducing the overall computation time compared to the Lu method.

### 3.5. Case Study of Docking Ring Detection in Spacecraft

To further test the application performance of our algorithm in docking tasks and verify its robustness in complex situations such as local occlusion, this section uses infrared images from the MEV-2 and IS 10-02 satellite docking process described in [34]. These images range in resolution from 300 × 300 to 400 × 400 pixels, with robotic arms added as occluding objects to simulate potential obstructions.

**Figure 8:**
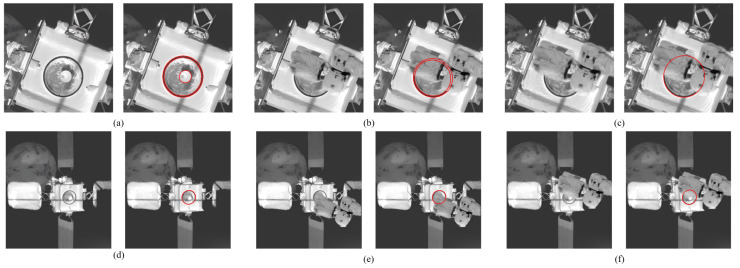
Examples of docking ring and ellipse detection results. (**a**–**c**) are infrared images captured by NFOV at 15 m, and (**d**–**f**) are infrared images captured by WFOV at 15 m. The left side of each subfigure is the original image with the robotic arm added, and the right side is the corresponding ellipse detection result.

Examples of the above data and experimental results are shown in Figure 8. It can be seen that in the infrared images captured by NFOV, the docking ring ellipse is larger. In this case, our algorithm can still reliably detect the docking ring ellipse even when occlusion exceeds 60%. In the infrared images captured by WFOV, the docking ring ellipse is relatively smaller. Our algorithm can still correctly detect the ellipse when the occlusion rate reaches 50%, demonstrating good adaptability to complex space environments. Additionally, our algorithm can complete the detection of the ellipse on the docking ring image within 50 ms, making it suitable for real-time docking tasks.

## 4. Conclusions

This paper proposes an efficient and robust ellipse detection method for spacecraft docking rings in complex scenes. We utilize the arc-support line segment method to extract arc segments and ensure the accuracy of detection through comprehensive score and weighted fitting. The hierarchical quadrant division is introduced to enhance the robustness of the algorithm. Experimental results show that the algorithm in this paper achieves good performance in five real-world datasets and real docking ring images, especially in terms of accuracy and F-measure. It can also detect the ellipse features of the inner and outer rings in real time in scenarios where more than half of the docking ring is occluded and can adapt well to complex space environments. Meanwhile, thanks to the multi-constraint combination and dynamic arc segment pruning strategy, the algorithm in this paper strikes a balance between accuracy and computing time and can meet the requirements of real-time docking tasks. Although the recall rate of the algorithm in this paper needs to be improved when dealing with small ellipses, overall, this algorithm provides an effective and reliable method for spacecraft docking ring detection with broad application prospects.

## Figures and Tables

**Table 1:** Key parameters and thresholds of the proposed algorithm.

Symbol	Description	Value
$\theta_{a}^{\mathrm{th}}$	Individual fitting threshold	$120^{{}^{\circ}}$
$\theta_{\mathrm{sum}}^{\mathrm{th}}$	Combined angle threshold	$80^{{}^{\circ}}$
$T_{\mathrm{pix}}$	pixel threshold	3 pixels
$\tau_{\theta }$	gradient orientation consistency threshold	$10^{{}^{\circ}}$
$T_{\mathrm{ratio}}$	edge validity threshold	80%
$T_{\mathrm{ang}}$	total coverage angle threshold	$150^{{}^{\circ}}$
$T_{\mathrm{den}}$	edge point density threshold	0.4
$\alpha_{1}$	scoring weights for edge density	1
$\alpha_{2}$	scoring weights for global coverage	1
$\alpha_{3}$	scoring weights for local continuity	1

**Table 2:** Experimental results of five different methods on five real datasets.

Dataset		Fornaciari’s [32]	Jia’s [21]	Lu’s [22]	Meng’s [23]	Ours
	accuracy	78.56	75.16	65.4	81.53	82.14
Satellite 1	recall	23.89	32.91	58.37	60.27	57.96
	F-measure	36.64	45.78	61.69	69.31	67.96
	accuracy	65.21	80.61	74.58	79.4	82.32
Satellite 2	recall	26.73	44.35	60.42	82.34	61.57
	F-measure	37.92	57.22	66.76	80.84	70.45
	accuracy	83.17	87.36	97.15	93.24	100
Smartphone	recall	69.85	67.36	91.92	87.32	92.65
	F-measure	75.93	76.07	94.46	90.18	96.18
	accuracy	46.72	62.76	60.4	69.82	70.93
PCB	recall	54.26	53.87	64.28	65.7	68.36
	F-measure	50.21	57.98	62.28	67.7	69.62
	accuracy	70.39	71.61	75.23	66.73	78.3
Prasad	recall	21.54	23.93	35.04	41.96	36.62
	F-measure	32.98	35.87	47.81	51.52	49.9

**Table 3:** The average detection time of five different methods on five real datasets (ms).

Dataset	Fornaciari’s [32]	Jia’s [21]	Lu’s [22]	Meng’s [23]	Ours
Satellite 1	6.78	5.11	34.83	5.74	29.64
Satellite 2	3.76	3.13	19.12	2.71	9.63
Smartphone	38.27	30.35	161.63	39.08	69.23
PCB	11.8	10.9	60.14	10.16	31.23
Prasad	5.63	5.28	43.52	6.14	18.54

## Data Availability

Data will be made available on request.
